# Biology of breast cancer in young women

**DOI:** 10.1186/s13058-014-0427-5

**Published:** 2014-08-27

**Authors:** Hatem A Azim, Ann H Partridge

**Affiliations:** 1 BrEAST Data Centre, Department of Medicine, Institut Jules Bordet, Université Libre de Bruxelles, Brussels, 1000 Belgium; 2 Department of Medicine, Dana Farber Cancer Center, Harvard Medical School, Boston, 02115-6013 MA USA

## Abstract

Breast cancer arising at a young age is relatively uncommon, particularly in the developed world. Several studies have demonstrated that younger patients often experience a more aggressive disease course and have poorer outcome compared to older women. Expression of key biomarkers, including endocrine receptors, HER2 and proliferation markers, appears to be different in younger patients and young women are more likely to harbor a genetic predisposition. Despite these differences, little research to date has focused on the biology of these tumors to refine prognosis, and potentially direct treatment strategies, which remain similar to those offered to older patients. Accumulating evidence suggests the differences in breast stroma in younger patients and changes that occur with pregnancy and breastfeeding likely contribute to the different biology of these tumors. Reproductive behaviors appear to impact the biology of tumors developing later in life. In addition, tumors arising during or shortly following pregnancy appear to exhibit unique biological features. In this review, we discuss our emerging understanding of the biology of breast cancer arising at a young age at both the pathologic and the genomic level. We elucidate the potential role of genomic signatures, the impact of pregnancy and breastfeeding on breast cancer biology, and how even current knowledge might advance the clinical management of young breast cancer patients.

**Electronic supplementary material:**

The online version of this article (doi:10.1186/s13058-014-0427-5) contains supplementary material, which is available to authorized users.

## Introduction

Breast cancer is predominantly a disease of aging, with only 5 to 7% of patients diagnosed below the age of 40 years in the developed world [1]. In less developed regions where population-based screening is not routine and populations are much younger on average, such as in Africa and the Middle East, a higher proportion of patients are diagnosed below the age of 40, reaching as high as 20% [2],[3]. Whether there are underlying genetic differences or environmental factors that would render women in Africa and the Middle East more prone to develop the disease at a young age is the subject of ongoing research [4].

Nevertheless, young age at diagnosis of breast cancer has emerged world-wide as an independent factor associated with higher risk of relapse and death in several large studies, even when more aggressive therapies are administered [5]–[9] (Table 1). Expression of key biomarkers, including endocrine receptors, HER2 and proliferation markers, appears to be different in younger patients. Recent studies have attempted to control for tumor molecular subtypes, recognizing that more aggressive subtypes are more common in younger women. Two studies suggested particularly worse outcomes in young patients compared to older women with luminal-B tumors [7],[9]. It was hypothesized that younger patients were not offered standard hormonal therapy until a decade ago, and compliance to hormonal therapy is lower in young patients [10]. However, a study of women who were untreated with systemic adjuvant therapy also demonstrated poorer outcomes of young luminal-B patients [9]. These collective findings suggest that tumors arising in younger patients may be more aggressive due to biological differences. In this review we discuss the biological features of breast cancer arising in young women, and the emerging relationship with reproductive behaviors, including pregnancy and breastfeeding, and their potential clinical implications.

**Table 1:** **Recent large studies investigating the impact of age on breast cancer prognosis**

	Young age, years (***n***)	Control age, years (***n***)	Outcome definition	Impact of young age on outcome^a^		Factors controlled in multivariate model
				Hazard ratio	95% CI	
Gnerlich *et al*. 2009 [5]	<40 (15,548)	≥40 (227,464)	BC-specific survival	1.39	1.34-1.45	T, N, grade, race, marital status, ER, PgR, local therapy
Fredholm *et al.* 2009 [6]	<35 (378)	50-69 (13,486)	BC-specific survival	1.76	1.36-2.28	T, N, grade, ER, multifocality, local and systemic therapy
Cancello *et al.* 2010 [7]	<35 (315)	35-50 (2,650)	BC-related event	1.7	1.33-2.18	T, N, grade, histology, ER, HER2, PgR, ki67 vascular invasion
Han *et al.* 2010 [8]	<35 (1,443)	40-50 (6,354)	Overall survival	30-34 years: 1.43 26–29 years: 1.97	1.18-1.74 1.48-2.62	T, N, ER, systemic therapy
Azim *et al.* 2012 [9]	≤40 (339)	>40 (2,562)	Relapse-free survival	1.34	1.10-1.63	T, N, grade, BC molecular subtype, systemic therapy

^a^In multivariate models. BC, breast cancer; CI, confidence interval; ER, estrogen receptor; *n*, number; N, nodal involvement; PgR, progesterone receptor; T, tumor size.

## Pathological features of tumors arising in young women

Recently, results of the largest prospective observation study evaluating the pathological features and outcome of women who were aged <40 years at diagnosis were reported [11]. This UK-based study included 2,956 patients diagnosed with breast cancer between 2000 and 2008. The median age at diagnosis was 36 years, and the majority had ductal histology (86.5%), and grade III (58.9%) tumors. Node-positive disease and multifocality were observed in 50.2% and 27%, respectively. One third of tumors were estrogen receptor (ER)-negative while one quarter were HER2-positive. Very similar results were observed among the first 399 patients evaluated in the Young Women’s Breast Cancer Study, which started in 2006 enrolling women aged 40 years or younger at diagnosis [12]. This study further demonstrated high rates of lymphovascular invasion and lymphocytic infiltration, in 34% and 24% of patients, respectively.

Several other retrospective studies have evaluated differences in pathological features according to age [5],[13]–[15]. Gnerlich and colleagues [5] conducted the largest analysis including more than 200,000 patients of whom nearly 15,000 were aged <40 years at breast cancer diagnosis from a Surveillance, Epidemiology, and End Results database. Young patients were more commonly diagnosed with larger tumors (*P* < 0.0001), nodal involvement (*P* < 0.0001), poorly differentiated tumors (*P* < 0.0001), and endocrine receptor-negative tumors (*P* < 0.0001). A population-based study from the California Cancer Registry, which included 5,605 patients aged <40 years at diagnosis, further showed higher expression of HER2 in the younger population [15]. Several other hospital-based studies confirmed the same findings [13],[14], underscoring that tumors diagnosed in younger patients have more aggressive pathological features.

## Pattern of breast cancer subtypes according to age

**Figure 1:**
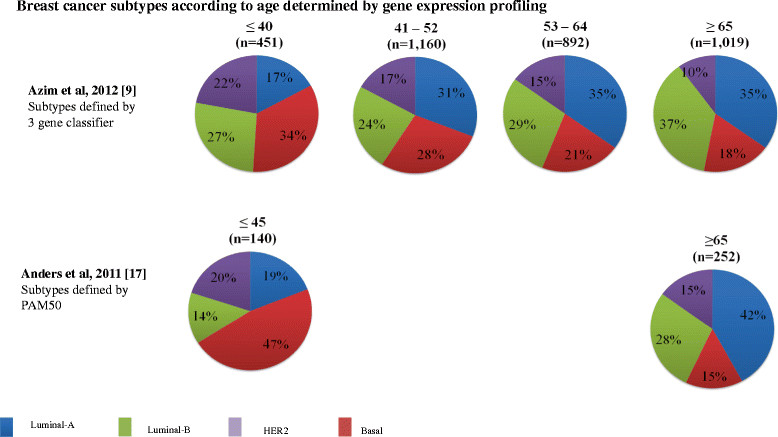
**Breast cancer subtypes.** Subtypes determined by gene expression profiling.

In recent years, breast cancer has been increasingly recognized as a heterogeneous disease with at least four subtypes: luminal-A, luminal-B, basal-like and HER2-enriched subtypes [16]. Figure 1 summarizes the two studies that have addressed the pattern of breast cancer molecular subtypes according to age using gene expression profiling. In the largest published study to date, Azim and colleagues [9] evaluated tumors of 3,522 patients, of whom 451 were aged ≤40 years at the time of breast cancer diagnosis. Young patients had a significantly higher proportion of basal-like tumors (34.3%) compared to 27.7%, 20.8% and 17.9% in patients aged 41 to 52, 53 to 64 and ≥65 years, respectively (*P* < 0.0001). A higher proportion of HER2-enriched tumors was also observed in young patients. On the other hand, young women were less likely to have luminal-A tumors (17.2%) compared to 30.7%, 35.1% and 35.4% in the other age groups (*P* < 0.0001).

**Figure 2:**
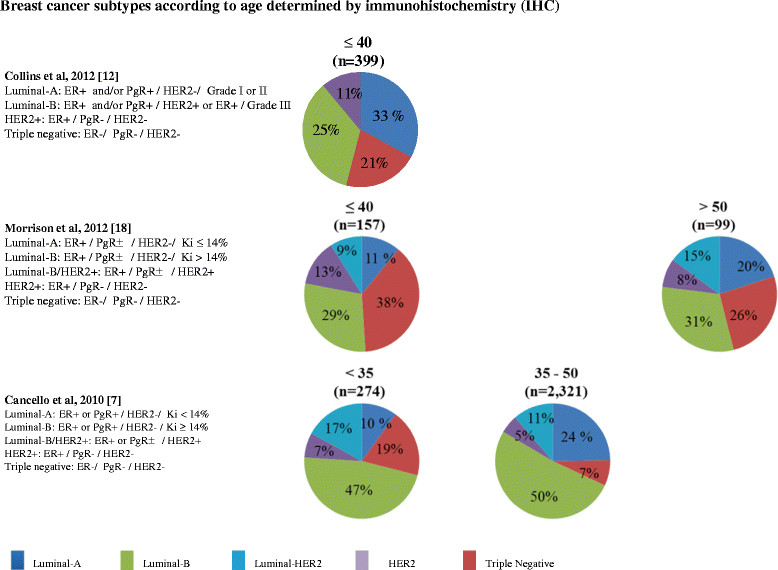
**Breast cancer subtypes.** Subtypes determined by immunohistochemistry. ER, estrogen receptor; PgR, progesterone receptor.

Other studies have addressed the question using immunohistochemical surrogates with variable definitions [7],[12],[17],[18] (Figure 2). This has resulted in different distributions of subtypes observed in the different studies. Of note, these studies were also hospital-based with potential for selection bias. The California Cancer Registry examined the differences in breast cancer subtypes according to age (<40, 40 to 49 and ≥50 years) but only based on the expression of ER and HER2 [15]. In line with other studies, there was a lower prevalence of ER-positive/HER2-negative tumors in younger patients (49% versus 63.9% versus 71.6%), but a high proportion of triple-negative tumors (22.8% versus 14.3% versus 11.7%), and also HER2 expression irrespective of ER status.

## Differences in pathological features and subtypes within premenopausal patients: does actual age matter?

In the previously discussed studies, different age cutoffs were used to define 'young age'. In addition, the term 'young age' has often been used synonymously with 'premenopausal' in evaluations of women with breast cancer, requiring further evaluation of whether differences exist within the premenopausal population according to actual age. In 2002, Colleoni and colleagues [13] published a large analysis including 1,427 premenopausal patients who were aged ≤50 years at the time of breast cancer diagnosis. They compared the expression of ER, progesterone receptor (PgR), and ki67 and other features by young age group (<35, 35 to 40, 40 to 45 and 45 to 50 years). Significant differences were observed according to age, with aggressive features more frequently observed in tumors arising in younger patients. Similar results were also reported by the Korean Breast Cancer Society registry, which included 9,885 premenopausal breast cancer patients aged ≤50 years at diagnosis [8].

In comparing groups of very young women, however, Collins and colleagues [12] did not find significant differences in histological features or the expression of ER, PgR and HER2 between patients aged ≤30 (n = 47), 31 to 35 (n = 111) and 36 to 40 (n = 241) years at breast cancer diagnosis in a prospective study, except for a trend of higher tumor necrosis in the youngest group (32% versus 14% and 21%, *P* = 0.06). A retrospective analysis of 500 patients who were aged <35 years at the time of diagnosis reported the same findings, albeit a modest higher prevalence of ER-negative (31% versus 23%), and highly proliferative tumors (ki67 > 30%; 59% versus 49%) among patients aged <30 and 30 to 34 years, respectively [19]. Collectively, these findings suggest that the younger the patient, the more aggressive the tumor features within the premenopausal population. Yet, it appears that differences are more subtle in women below 35 or 40 years.

## Molecular profiling of breast cancer in young women

### Gene expression differences

In 2008, Anders and colleagues [20] published one of the first attempts to describe the biology of breast cancer in young women using gene expression profiling. In this analysis, which included 200 patients in the young group (≤45 years) and 211 patients in the control group (≥65 years), a higher probability of phosphatidylinositide 3-kinase (PI3K; *P* = 0.006) and Myc (*P* = 0.03) pathway deregulation was observed in tumors arising in younger patients. However, this analysis was not adjusted for potential differences in breast cancer molecular subtypes as well as other known prognostic factors. Subsequently, a similar analysis was performed by the same group with appropriate adjustment for molecular subtypes among other features [17], using two publicly available datasets; the first including 48 patients aged ≤45 years and 144 patients ≥65 years, and the second including 92 patients ≤45 years and 108 patients ≥65 years. As expected, younger patients in their datasets had more basal-like tumors but, after adjustment for subtype differences, no distinct molecular aberrations were found that were related to age.

More recently, Azim and colleagues [9] conducted a pooled gene expression analysis on two datasets including 1,188 and 2,334 patients. The aim was to evaluate the association between patients’ age and nearly 50 genes that were identified based on literature search to be related to early-onset breast cancer. The analysis was adjusted for differences in breast cancer molecular subtype, histological grade, tumor size, and nodal status. Results on the first dataset (n = 1,188, ≤40 years = 191) showed that, independent of subtype, grade and stage, younger patients have higher expression of *RANK-ligand* (*P* < 0.0001), *c-kit* (*P* < 0.001), in addition to mammary stem cell (*P* < 0.0001) and luminal progenitors and *BRCA1* mutation signatures (*P* = 0.007). In addition, there was more disruption of the mitogen-activated protein kinase and PI3K pathways (*P* < 0.0001) and lower expression of *BRCA1* (*P* = 0.003) and several apoptosis-related genes, particularly *FAS* (*P* = 0.03). The very same findings were reproduced in an independent dataset that included 2,334 patients, of whom 260 were aged ≤40 years. At a glance these results appear in direct conflict with those of Anders and colleagues [17]. However, the Anders analysis included four times fewer patients and utilized an unbiased approach in searching for genes associated with age, which requires a relatively high number of patients, especially with adjustment for several confounders and multiple comparisons.

The results by Azim and colleagues suggest interesting insights into the biology of early onset breast cancer. The high *BRCA1* mutation signature expression is consistent with the known relatively high prevalence of *BRCA1* mutations in younger patients [21],[22]. Patients with *BRCA1* mutations are commonly diagnosed with basal-like tumors [23]; earlier work suggested that luminal progenitors appear to be the cell of origin of these tumors and are regulated by c-kit [24]. The high expression of the *BRCA1* mutation signature, luminal progenitors and c-kit in younger patients all may suggest why young women tend to develop basal-like tumors at higher frequencies. The high expression of RANKL (Receptor activator of nuclear factor kappa-B ligand) is also intriguing given RANKL is known to stimulate osteoclastogenesis and targeting RANKL has been shown to reduce risk of osteoporosis and related skeletal events secondary to bone metastases [25]. RANKL has also emerged as a PgR-regulated gene that is involved in the expansion of mammary stem cells, increasing their proliferation and protecting them from undergoing apoptosis [25]. In young women, the normal breast is enriched with an immature mammary cell population (that is, stem cells and progenitors), which increases during pregnancy and breastfeeding, an effect that has been shown to be mainly regulated by RANKL [26]. In preclinical breast cancer models, RANKL inhibition arrested progestin-induced cancer and reduced the mammary stem cell component [27]. Thus, RANKL appears to be a potentially relevant breast cancer target beyond its established role in managing bone metastases.

### Prognostic genomic signatures in young breast cancer patients

Currently several genomic tests are available to improve prognostication and aid decision making in the adjuvant setting [16]. This includes Oncotype Dx®, Mammaprint®, Endopredict, PAM50, Breast Cancer Index and many others [28]. They add prognostic information to classic prognostic variables in patients with ER-positive tumors and appear to distinguish reliably between patients at low and high risk of recurrence [28]. They are increasingly integrated in standard clinical practice, yet there have been concerns about whether they carry the same prognostic value in young women given these signatures were mainly developed using populations of postmenopausal women.

The initial work by the Dutch group on MammaPrint® including 295 patients, only 63 (21%) of whom were younger than 40 years, revealed 52/63 young patients (82%) were classified as high risk [29]. The same was observed in earlier studies with Oncotype Dx®, where only 59 out of 668 patients were aged less than 40 years, yet the majority had a high risk score (33/59 young patients; 56%) [30]. This was somewhat higher than the proportions in the high risk group in patients aged 40 to 50 (29%), 50 to 60 (25%) and >60 years (21%). The other signatures were also largely developed using populations of older patients and hence it is hard to extrapolate from these studies the value of genomic signatures in the young population.

A pooled gene expression analysis recently addressed the prognostic value of three signatures according to age: GENE70 (the microarray version of MammaPrint®), the genomic grade index and GENE76 [9]. In an analysis including 755 patients with ER-positive disease, of whom 87 were aged ≤40 years, each of the genomic signatures was significantly associated with disease-free survival and added significant prognostic information to the clinical risk classifier, Adjuvant Online. The prognostic value was the same across all age groups, suggesting that genomic signatures can add prognostic information in younger as well as older women with breast cancer.

### Pattern of mutations in young breast cancer patients

Several recent studies have reported on the landscape of somatic mutations in breast cancer using next generation sequencing [31]–[33]. Point mutations have been observed in *TP53* and *PIK3CA* genes, accounting for nearly 25% of cases. However, very little is known regarding the pattern of somatic mutations in younger women. Stephens and colleagues [33] conducted whole genome sequencing of 100 breast tumors but found no correlation between total number of somatic base substitution and age at diagnosis in both ER-positive (*P* = 0.33) and ER-negative (*P* = 0.14) tumors.

Recently, the pattern of hot spot somatic mutations using Sequenom was evaluated in 167 young breast cancer patients (mean age of 36 years), of whom 54 were diagnosed during pregnancy [34]. A total of 84 mutations in 19 genes were evaluated, including 29 different mutations of *PIK3CA* (94% of known mutations), and 7 and 6 mutations for *ERBB2* and *TP53*, respectively. No differences were observed between the pregnant and non-pregnant groups. While this study lacked a control group, the prevalence of mutations particularly in *PIK3CA* appeared to be in line with their known prevalence in older women: approximately 23%. Only 5% of patients had a *TP53* mutation, although it should be noted that only 12% of known *P53* mutations were explored in this study. No *ERBB2* mutations were observed at all.

Regarding germline mutations, *BRCA1/2* mutations are the most common, accounting for up to 40% of familial breast cancer [35]. In a large analysis including 3,345 patients who were aged ≤50 years at the time of breast cancer diagnosis, 7% of patients had a *BRCA1* mutation [21]. However, *BRCA1* carriers were significantly younger (mean age 41.9 versus 44.1, *P* < 0.001), and had more ER-negative (84.1% versus 38.1%, *P* < 0.001) and HER2-negative (93% versus 79%, *P* < 0.001) tumors.

Data on other familial breast cancer syndromes in young women are very scarce. CHEK2*1100delC is another germline mutation that has been described to occur more commonly in younger patients. A recent study from Denmark evaluating 25,571 patients found that 1.8% were CHEK2*1100delC heterozygous [36]. These patients were younger and were more likely to be premenopausal and have ER-positive disease (all *P* < 0.001).

The fact that women with familial breast cancer syndromes appear to develop the disease more frequently at an earlier age adds further complexity to the biological make-up of breast cancer in young women. Further research to elucidate the triggers for the development of disease in this high risk young population in particular is clearly warranted.

## Impact of pregnancy/breastfeeding on breast cancer biology

### Reproductive behavior and biology of subsequent breast cancer

Decades ago it was shown that pregnancy increases breast cancer risk in the short term but has a long-term protective effect [37]. More recently, several large studies have evaluated the relationship between different reproductive behaviors and not only the risk but also the phenotype of subsequent breast cancer [38]–[42] (Table 2). Recent studies suggest a protective effect of parity on the development of ER-positive tumors at the expense of a relatively higher proportion of patients diagnosed with triple-negative disease particularly in the absence of breastfeeding. On the other hand, breastfeeding appears to be protective against triple-negative breast cancer [38],[40],[42]. This is also true for *BRCA1* carriers, in whom breastfeeding for 1 or 2 years was shown to be associated with a 32% and 49% reduction in breast cancer risk, respectively [43]. Breastfeeding was protective for both early- and late-onset cancers in this high risk population.

**Table 2:** **Recent large studies investigating the impact of pregnancy and breastfeeding on the risk of developing breast cancer according to biology**

	Population	Number	Impact of parity on breast cancer risk according to subtype	Impact of breastfeeding on breast cancer risk according to subtype
Shinde *et al.* 2010 [38]	MD Anderson	2,473	Increase TNBC risk	Reduce TNBC risk
Palmer *et al.* 2011 [39]	African American	457	Reduce ER + BC risk	Reduce TNBC risk
Redondo *et al.* 2012 [40]	Spanish	501		Reduce TNBC risk
Chung *et al.* 2013 [41]	Korean	6,952	Reduce ER + BC risk	
Li *et al.* 2013 [42]	American	1,962 (<45 years)	Reduce ER + BC risk	Reduce TNBC risk

BC, breast cancer; ER+, estrogen receptor-positive; TNBC, triple-negative breast cancer.

The biological explanation of the effect of pregnancy and breastfeeding on breast cancer risk is poorly understood. Russo and colleagues [44] compared gene expression profiles of microdissected epithelial cells from normal breast tissue of 41 parous and 8 nulliparous post-menopausal breast cancer patients with those of 18 parous and 7 nulliparous post-menopausal women without breast cancer. They found that parous non-cancer patients had unique gene expression patterns including differential expression of apoptosis-related genes and others related to cell cycle and cell signaling. This suggested that pregnancy may induce a signature that protects from developing breast cancer. However, this study was based on few study subjects, and lacked long-term follow-up to confirm that the parous non-cancer group did not develop subsequent breast cancer. Asztalos and colleagues [45] studied gene expression patterns in human breast after pregnancy to try to elucidate the bidirectional effect of pregnancy on cancer risk. They grouped 52 young women (median age 29 years) as nulliparous, recently pregnant (that is, 0 to 2 years since last pregnancy) and distantly pregnant (that is, 5 to 10 years since last pregnancy) and evaluated a panel of 64 genes related to immune, angiogenesis, extracellular remodeling and hormone signaling. The parous groups had lower expression of ERα, PgR, and HER2 but higher expression of ERβ and inflammation-associated genes. No considerable differences were observed between the recently and distantly pregnant groups. A more recent preclinical study was able to show that parity downregulates Wnt/Notch signaling and suppresses progenitor cells, suggesting that this could be a potential mechanism explaining the long-term protective effect of pregnancy [46].

### Pregnancy-associated breast cancer

Several studies and a recent meta-analysis have shown that patients diagnosed with breast cancer during or shortly after pregnancy have poor prognosis, particularly those diagnosed shortly after pregnancy [47]. Clearly, delays in diagnosis in this unique population has some effect, although it is also plausible that the hormonal milieu and the massive increase of female sex hormones during pregnancy can modulate the breast microenvironment, and consequently stimulate aggressive tumor growth. An alternative hypothesis is that the processes of breast involution that occurs following delivery, similar to wound healing where angiogenesis, inflammation and extracellular matrix alterations are activated, results in more aggressive breast cancer biology [48].

Schedin and colleagues [49],[50] have developed a preclinical model investigating the impact of post-partum mammary involution on breast cancer initiation and progression. In this model, tumors developing in an involuting breast were larger, greater in number and had a higher proliferation index compared to those developing in a nulliparous breast. They reported that this phenomenon was related to high deposition of collagen and expression of Cox-2 in both the tumor and the involuting breast. Furthermore, inhibition of Cox-2 by a Cox-2 inhibitor resulted in reductions in the size of the tumors arising in the involuting breast. This study provided a proof-of-concept that pregnancy alters the breast microenvironment, which could subsequently impact tumor development and tumor biology.

In patients diagnosed during pregnancy, several analyses of clinical data have not revealed different expression of key biomarkers like ER and HER2 compared with non-pregnant age-matched breast cancer patients [51]–[53]. A recent gene set enrichment analysis that included 54 pregnant and 113 age- and stage-matched non-pregnant breast cancer patients revealed that tumors diagnosed during pregnancy were associated with activated signaling pathways like the serotonin receptor pathway and G-protein-coupled receptor pathway [34]. There was also high expression of relevant cancer targets related to PD1/PDL1, SRC, insulin growth factor and Wnt/β-catenin. The expression of these genes in normal breast increased steadily over the course of pregnancy in a mouse model, underscoring the potential role of the breast microenvironment during pregnancy on the tumor phenotype. While it is difficult to validate these experiments clinically given the rarity of the disease and complexities of enrolling pregnant women into clinical trials, these collective findings underline that changes that occur during pregnancy and in the post-partum period likely impact the biology of breast cancer development in young women. These changes are most likely induced by the hormonal and inflammatory changes in these periods, and the resulting perturbation of the breast microenvironment. However, current data do not confirm that such effects play a key role in driving carcinogenesis and tumor biology.

### Future directions

There is clear evidence that breast cancer arising at a young age is more aggressive and has potentially unique biological features and that events occurring during the childbearing period, including pregnancy and breastfeeding, impact on not only breast cancer risk but also breast cancer phenotype and biology. Nevertheless, to date, management strategies are often the same irrespective of age and hence there is a need to adapt a biology-driven approach to refine treatment for younger breast cancer patients [54].

These patients have a relatively high risk of relapse and hence it would be vital to integrate novel genomic tools to refine the treatment-decision process. Genomic tests could guide not only those patients who derive little benefit from adjuvant chemotherapy but also those who might be more suitable for extended adjuvant therapy, a strategy that has proven effective in recent studies [55],[56]. Three genomic signatures, PAM50, Endopredict and Breast Cancer Index, were shown to reliably determine risk of recurrence beyond 5 years [57]–[59]. Considering that nearly 40 to 50% of young ER-positive patients relapse after 5 years [11], they could serve as a tool to identify those that would derive higher benefit from extended adjuvant therapy. This approach needs to be validated in clinical trials.

Based on the results obtained from the large gene expression analysis showing high expression of RANKL in younger patients, a preoperative window trial is currently ongoing to evaluate the impact of denosumab, a RANKL inhibitor, on the biology of breast cancer in young women (D-BEYOND; NCT01864798). This study could potentially define a role for denosumab in future management of young patients.

Another ongoing study in patients with pregnancy-associated breast cancer is investigating the role of Cox-2 inhibition (NCT01881048). Patients in the post-partum period will receive one week of celecoxib to evaluate its effect on the proliferation marker ki67 prior to surgical intervention.

Poly ADP ribose polymerase inhibitors are emerging as very promising drugs in managing patients with *BRCA1* or *BRCA2* mutations [60]. A study in the adjuvant setting is currently evaluating the value of 1 year of adjuvant olaparib in *BRCA1* mutated patients (NCT02032823) and a relatively large number of young patients will likely enroll.

In light of the emerging evidence that young women with luminal-B tumors have particularly disparate outcomes, and in consideration of the high deregulation of the PI3K pathway in tumors arising in young patients [9],[20] and its vital role in endocrine resistance [61], targeting the PI3K pathway is an approach that is worth investigating further in young breast cancer patients. Targeting premature mammary cell subpopulations that appear to be abundant in younger patients is also worth exploring with agents such as Notch inhibitors, a novel class targeting stem cells-like features [62].

Finally, better characterization of somatic mutations occurring in tumors arising in young women using next generation sequencing could further identify key driver mutations that can be targeted in this challenging disease. These strategies and others underscore that a lot of work is still required to elucidate the biology and consequently improve the outcomes of young women with breast cancer.

## Authors’ original submitted files for images

Below are the links to the authors’ original submitted files for images. Authors’ original file for figure 1 Authors’ original file for figure 2

## Abbreviations

ER: Estrogen receptor
RANKL: Receptor activator of nuclear factor kappa-B ligand
PgR: Progesterone receptor
PI3K: Phosphatidylinositide 3-kinase
