# Supplementary figures and images for: Biology of breast cancer in young women
Source: Breast Cancer Res. 2014 Aug 27;16:427. doi: 10.1186/s13058-014-0427-5 (PMC4303229; doi:10.1186/s13058-014-0427-5)

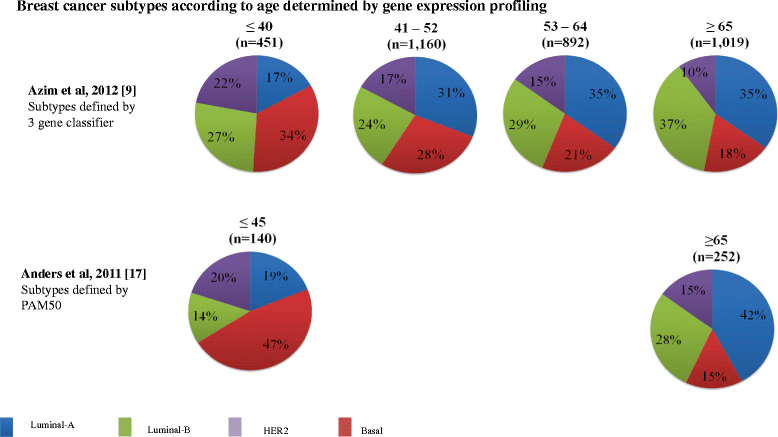

Supplement: Supplementary file 1 — Authors’ original file for figure 1 [file 13058_2014_427_MOESM1_ESM.gif]

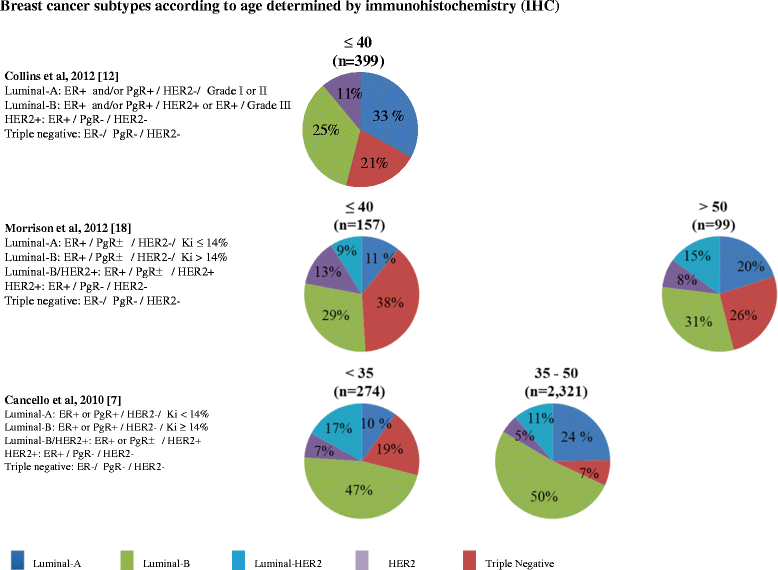

Supplement: Supplementary file 2 — Authors’ original file for figure 2 [file 13058_2014_427_MOESM2_ESM.gif]
